# Supplementary material for: Efficacy of Virtual Reality Interventions for Motor Function Improvement in Cerebral Palsy Patients: Systematic Review and Meta-Analysis
Source: J Clin Med. 2025 Nov 26;14(23):8388. doi: 10.3390/jcm14238388 (PMC12692755; doi:10.3390/jcm14238388)
Supplement: Supplementary file 1 [file jcm-14-08388-s001.zip › jcm-3912964-supplementary.pdf]

Supplementary Table S1: Study Inclusion In Primary Meta-Analysis.

Category/Study	Design/Analysis Type	N	Age (yr)	Technology/Comparison	Effect Size [95% CI]	P-value	I²	Included?/Interpretation
**INDIVIDUAL STUDIES SCREENED (n=16, N=397):**								
Roberts et al. 2025 [43]	RCT (blinded)	33	5-13	Mixed robotic systems; VR+CIMT vs CIMT	—	—	—	✓ Included
Saussez et al. 2023 [44]	Non-inferiority RCT	40	5-18	Semi-immersive; VR+HABIT vs HABIT	—	—	—	✓ Included
Roostaei et al. 2023 [14]	Single-case experimental	8	7-18.4	Custom Kinect; Pre-post	—	—	—	✗ Non-RCT design
Fu et al. 2022 [45]	RCT	60	6-11	Lokomat (robotic); VR vs Standard PT	—	—	—	✓ Included
Roberts et al. 2020 [46]	Pre-post	32	5-15	Robotic exoskeleton; Pre-post	—	—	—	✗ Non-RCT design
Bortone et al. 2020 [47]	RCT Crossover	8	NR	Immersive VR+haptic; VR vs Conventional	—	—	—	✗ Incomplete data (crossover)
Decavele et al. 2020 [48]	RCT Crossover	32	6-15	Non-immersive gaming; VR vs Standard PT	—	—	—	✗ Incomplete data (crossover)
Gagliardi et al. 2018 [49]	Pre-post pilot	16	7-16	GRAIL immersive; Pre-post	—	—	—	✗ Non-RCT design
El-Shamy et al. 2018 [50]	RCT	30	6-8	Armeo Spring (robotic); VR vs Conventional	—	—	—	✓ Included
Yoo et al. 2017 [35]	Non-randomized crossover	10	7-15	Commercial gaming; VR vs Standard	—	—	—	✗ Non-RCT design
Acar et al. 2016 [51]	RCT	30	6-15	Nintendo Wii gaming; VR vs NDT	—	—	—	✓ Included
Preston et al. 2016 [52]	RCT	15	8-18	Custom VR; VR vs Standard	—	—	—	✗ Incomplete data (baseline only)
Lazzari et al. 2015 [53]	RCT	20	7-12	Nintendo Wii; VR+tDCS vs VR alone	—	—	—	✗ Both groups received VR
Grecco et al. 2015 [54]	RCT	20	5-12	Nintendo Wii; VR+tDCS vs tDCS alone	—	—	—	✗ Confounded comparison
Preston et al. 2014 [55]	Feasibility study	11	8-18	Custom VR; Pre-post	—	—	—	✗ Non-RCT feasibility
Rostami et al. 2012 [56]	RCT	32	5-12	Gaming system; VR vs Standard	—	—	—	✗ Incomplete data (no SDs)
STUDIES TOTAL	16 studies	397	5-18.4	Multiple technologies/comparisons	—	—	—	5 RCTs included, 11 excluded
**POOLED META-ANALYSIS RESULTS (k=5 RCTs, N=190, ES=40):**								
OVERALL EFFECT								
VR vs Control (All)	Pooled effect (k=5)	190	5-18	40 effect sizes across all studies	0.41 [0.16, 0.66]	0.001	74%	Moderate effect favoring VR
BY TECHNOLOGY TYPE								
Robotic/Exoskeleton vs Control	Subgroup (k=3)	90	5-13	17 effect sizes	1.00 [0.37, 1.63]	0.002	90%	Large effect favoring robotic VR
Commercial Gaming vs Control	Subgroup (k=2)	30	6-15	7 effect sizes	0.38 [0.08, 0.68]	0.013	15%	Small-moderate effect
Custom VR vs Control	Subgroup (k=2)	38	5-18	14 effect sizes	0.01 [-0.16, 0.18]	0.905	0%	No significant effect
Mixed Technologies vs Control	Subgroup (k=1)	32	5-13	2 effect sizes	-0.08 [-0.69, 0.52]	0.789	32%	No significant effect
Test for subgroup differences	Heterogeneity test	—	—	Q=29.00, df=3	—	<0.001	—	Technology type is significant moderator
BY COMPARISON TYPE								
VR vs Standard Care	Subgroup (k=5)	190	5-18	17 effect sizes	0.83 [0.50, 1.16]	<0.001	48%	Large effect favoring VR
VR vs Active Control	Subgroup (k=5)	190	5-18	23 effect sizes	0.09 [-0.11, 0.28]	0.372	40%	No significant difference
Test for subgroup differences	Heterogeneity test	—	—	Q=61.79, df=1	—	<0.001	—	Comparison type is significant moderator
BY OUTCOME DOMAIN								
Upper Limb Function	Subgroup (k=4)	130	5-18	20 effect sizes	0.59 [0.30, 0.88]	<0.001	77%	Moderate-large effect
Gross Motor Function	Subgroup (k=1)	60	6-11	6 effect sizes	1.52 [0.99, 2.05]	<0.001	38%	Very large effect
Walking Capacity	Subgroup (k=1)	98	—	2 effect sizes	0.14 [-0.25, 0.53]	0.479	0%	No significant effect
Functional Activities	Subgroup (k=3)	100	—	12 effect sizes	0.13 [-0.18, 0.44]	0.413	55%	No significant effect
Balance	Insufficient data	0	—	0 effect sizes	Insufficient data	—	—	Unable to analyze
Test for subgroup differences	Heterogeneity test	—	—	Q=81.25, df=4	—	<0.001	—	Outcome domain is significant moderator
BY AGE GROUP								
(<6 years)	Subgroup (k=1)	60	<6	12 effect sizes	0.98 [0.43, 1.52]	<0.001	87%	Large effect
School-age (6-12 years)	Subgroup (k=3)	100	6-12	21 effect sizes	-0.01 [-0.17, 0.15]	0.903	0%	No significant effect
Test for subgroup differences	Meta-regression	—	—	Q=26.36, df=1	—	<0.001	—	Age is significant moderator (slope=-0.236/yr, R^2=0.18)
DOSE-RESPONSE ANALYSIS								
Optimal dose (30-40 hours)	Subgroup (k=2)	90	—	Peak response zone	~0.72 (peak effect)	—	—	Non-linear inverted U-shaped relationship
High dose (>50 hours)	Subgroup (k=2)	70	—	Beyond optimal range	~0.00 (diminishing returns)	—	—	Diminishing returns beyond 50 hours
Quadratic meta-regression	Non-linear model (k=5)	190	—	Dose²-effect relationship	β_2=-0.0003	0.001	R^2=0.14	Significant non-linear dose-response curve

Abbreviations: *k=number of studies; N=number of participants; ES=effect sizes; SMD=standardized mean difference; CI=confidence interval; I²=heterogeneity; VR=virtual reality; RCT=randomized controlled trial; CIMT=constraint-induced movement therapy; HABIT=hand-arm bimanual intensive therapy; PT=physiotherapy; NDT=neurodevelopmental therapy; tDCS=transcranial direct current stimulation; NR=not reported.*

Supplementary Table S2: Detailed Moderator Analysis Results.

Moderator Variable	Subgroup/Level	N Studies	N Participants	N Effect Sizes	SMD [95% CI]	p-value	I^2 (%)	Q-test (between)	R^2	Significance
**TECHNOLOGY TYPE:**										
VR Technology	Robotic/Exoskeleton	3	90	17	1.00 [0.37, 1.63]	0.002	90	Q=29.00, df=3, p<0.001	N/A	Large effect; clinically meaningful
	Commercial Gaming	2	30	7	0.38 [0.08, 0.68]	0.013	15			Small-moderate effect
	Custom VR	2	38	14	0.01 [-0.16, 0.18]	0.905	0			No significant effect
	Mixed Technologies	1	32	2	-0.08 [-0.69, 0.52]	0.789	32			No significant effect
**EXOSKELETON vs NON-EXOSKELETON:**										
Platform Type	Exoskeleton-based	3	122	19	0.85 [0.31, 1.38]	0.002	89	Q=20.39, df=1, p<0.001	N/A	Large effect
	Non-exoskeleton	4	68	21	0.16 [-0.01, 0.33]	0.068	0			Minimal effect
**COMPARISON TYPE:**										
Control Group	VR vs Standard Care	5	190	17	0.83 [0.50, 1.16]	<0.001	48	Q=61.79, df=1, p<0.001	N/A	Large effect; VR superior
	VR vs Active Control	5	190	23	0.09 [-0.11, 0.28]	0.372	40			No difference; VR not superior
**AGE (CONTINUOUS)**										
Meta-regression	Continuous (per year)	4	160	4 study-level	Slope: -0.236, Intercept: 2.37	<0.001	N/A	N/A	0.18	Effect decreases 0.24 SD per year
**AGE (CATEGORICAL):**										
Age Group	(<6 years)	1	60	12	0.98 [0.43, 1.52]	<0.001	87	Q=26.36, df=1, p<0.001	N/A	Large effect in young children
	School-age (6-12 years)	3	100	21	-0.01 [-0.17, 0.15]	0.903	0			No effect in older children
**DOSE-RESPONSE (LINEAR):**										
Linear model	Per hour increase	5	190	5 study-level	Slope: 0.002	0.638	N/A	N/A	0.00	No linear relationship detected
**DOSE-RESPONSE (QUADRATIC):**										
Quadratic model	Non-linear (U-shaped)	5	190	5 study-level	Intercept: 0.36, Quadratic: -0.0003	<0.001	N/A	N/A	0.14	Inverted U-shape confirmed, Optimal dose: 30-40 hours, Peak SMD≈0.72 at 37h
**THRESHOLD ANALYSIS:**										
Dose threshold	<50 hours	5	120	24	0.66	<0.001	Moderate	Threshold: 50h	N/A	Moderate effect below threshold
	≥50 hours	2	70	16	-0.00	1.000	Low	Difference: -0.66, p<0.001		No effect above threshold

Supplementary Table S3: Multivariable Meta-Regression Analysis.

Model Component	Variable/Statistic	Coefficient (β) / Value	Standard Error	t-value	P-value	95% CI Lower	95% CI Upper	Interpretation	Clinical Meaning
**FIXED EFFECTS:**									
Predictor	Intercept	2.37	0.48	4.93	<0.001	1.43	3.31	Baseline effect (age 0, non-robotic, 0h)	Very large theoretical benefit at birth
	Age (continuous, per year)	-0.24	0.04	-6.00	<0.001	-0.31	-0.16	Each year reduces effect by 0.24 SD	Age 4y: SMD≈1.4; Age 10y: SMD≈0.0
	Technology: Robotic (vs non-robotic)	0.78	0.24	3.25	0.002	0.30	1.27	Robotic adds 0.78 SD benefit	3× larger effect than gaming/custom
	Total Hours (continuous)	0.01	0.01	1.87	0.062	0.00	0.03	Marginal linear dose effect (NS)	Non-linear dose-response confirmed
**MODEL FIT:**									
Variance Explained	R^2 (proportion)	0.33	-	-	-	-	-	Model explains 33% of heterogeneity	Age + technology account for 1/3 variance
Residual Heterogeneity	Residual I^2	49%	-	-	-	-	-	49% variance remains unexplained	Unmeasured moderators present
Baseline Heterogeneity	Baseline I^2	74%	-	-	-	-	-	Starting heterogeneity before model	Model reduces I^2 by 25 percentage points
Model Selection	AIC (Akaike)	118.4	-	-	-	-	-	Complexity-penalized fit index	Lower values indicate better fit
Sample Size	N studies	5	-	-	-	-	-	Study-level meta-regression	Limited power for complex models
Degrees of Freedom	df	1	-	-	-	-	-	5 studies - 4 parameters	Minimal df; caution with overfitting
**PREDICTOR SIGNIFICANCE:**									
Significance Summary	Age	Highly significant	Strong effect	-6.00	<0.001	Robust	Robust	Strongest moderator	Critical patient selection factor
	Robotic Technology	Significant	Moderate-large	3.25	0.002	Robust	Robust	Second strongest	Technology choice matters
	Total Hours	Non-significant	Marginal	1.87	0.062	Weak	Weak	Linear dose insufficient	Use quadratic dose model instead
**CLINICAL IMPLICATIONS:**									
Treatment Selection	Patient Age	Critical factor	Decreases 0.24/y	Large	Essential	High confidence	High confidence	Younger = better	Target preschool children
	VR Platform	Critical factor	Robotic superior	Large	Essential	High confidence	High confidence	Exoskeletons preferred	Especially for older children
	Intervention Dose	Minor factor	Linear NS	Small	Optional	Low confidence	Uncertain	Non-linear pattern	Use 30-40h sweet spot from quadratic
Model Limitations	Unexplained Variance	67% (residual I^2=49% / baseline I^2=74%)	-	-	-	-	-	Other moderators exist	Setting, severity, frequency not in model

Supplementary Table S4: Heterogeneity Estimator Comparison.

Estimator Method	τ² (Between-study variance)	I² (%)	H²	Pooled SMD	95% CI Lower	95% CI Upper	CI Width	Precision vs DL	Key Advantage	Key Disadvantage	Recommended When	Performance Rating	Computational Complexity
DerSimonian-Laird (DL)	0.42	74	3.85	0.41	0.16	0.66	0.50	Reference (100%)	Most widely used; simple	Underestimates τ² in small k	Large meta-analyses (k>10)	Good for large k	Low (closed-form)
REML (Restricted Maximum Likelihood)	0.36	72	3.57	0.43	0.19	0.68	0.49	+2% narrower	Less biased in small k; better CI	Slightly complex	Small meta-analyses (k<10)	Best for k=5	Medium (iterative)
Paule-Mandel (PM)	0.39	73	3.70	0.44	0.19	0.69	0.50	Equal to DL	Robust to outliers	Unstable in very small k	Moderate heterogeneity	Good for k=5	Medium (iterative)
RANGE ACROSS METHODS	0.36-0.42 (Δ=0.06)	72-74% (Δ=2%)	3.57-3.85	0.41-0.44 (Δ=0.03)	0.16-0.19	0.66-0.69	0.49-0.50	Within 2%	High consistency	No major issues	Any valid choice	All acceptable	All feasible
CONSISTENCY ASSESSMENT	Very high	Very high	High	Very high (Δ<0.05)	Moderate	Moderate	Very high	Minimal difference	Robust to method	No method artifacts	Results trustworthy	Validated	Not a concern
RECOMMENDATION FOR k=5	REML preferred	REML optimal	-	Use REML (0.43)	Use REML	Use REML	REML narrowest	+2% gain	Best small-sample properties	None for k=5	This meta-analysis	★★★★★	Standard software
SENSITIVITY CONCLUSION	Insensitive (max Δ=0.06)	Insensitive (max Δ=2%)	Stable	Robust (max Δ=0.03 SD)	All CIs overlap	All CIs overlap	Nearly identical	Method choice irrelevant	Primary results validated	No methodological artifact	High confidence in SMD≈0.41-0.44	Confirmed	Results trustworthy
HETEROGENEITY INTERPRETATION	Substantial (τ²>0.30)	High (I²>70%)	Large (H²>3)	Random-effects essential	Wide prediction interval	Moderators needed	Caution generalizing	Explore sources	Age + technology explain 33%	67% variance unexplained	Context-dependent effects	Not one-size-fits-all	Personalize interventions

Supplementary Table S5: Risk of Bias Assessment Using Cochrane Risk of Bias 2.0 (RCTs) and Adapted Criteria (Non-randomized Studies).

Study	Study Design	Randomization Process	Deviations from Intended Interventions	Missing Outcome Data	Measurement of Outcome	Selection of Reported Result	Overall Risk of Bias	Justification
Roberts et al. 2025 [43]	RCT (blinded)	Low risk	Some concerns	Low risk	Low risk	Low risk	Some concerns	Low risk in most domains, some concerns due to lack of participant/therapist blinding
Saussez et al. 2023 [44]	Non-inferiority RCT	Low risk	Some concerns	Low risk	Low risk	Low risk	Low risk	Well-conducted RCT with adequate methodology and reporting
Roostaei et al. 2023 [14]	Single-case experimental	Not applicable	Not applicable	Low risk	Some concerns	Low risk	Some concerns	Appropriate single-case methodology but limited by design constraints
Fu et al. 2022 [45]	RCT	Some concerns	Some concerns	Low risk	High risk	Some concerns	High risk	Multiple domains with concerns, lack of blinding and methodological details
Roberts et al. 2020 [46]	Pre-post (single group)	Not applicable	Not applicable	Some concerns	High risk	Some concerns	High risk	Single-group design limits causal inference, unblinded assessment
Bortone et al. 2020 [47]	RCT Crossover (pilot)	Some concerns	Some concerns	Some concerns	Some concerns	Low risk	Some concerns	Pilot study with adequate registration but methodological limitations
Decavele et al. 2020 [48]	RCT Crossover	Low risk	Some concerns	Some concerns	Some concerns	Low risk	Some concerns	Adequate randomization but concerns about blinding and missing data
Gagliardi et al. 2018 [49]	Pre-post (pilot)	Not applicable	Not applicable	Low risk	Some concerns	Some concerns	Some concerns	Well-conducted pilot but limited by single-group design
El-Shamy et al. 2018 [50]	RCT	Low risk	Some concerns	Low risk	Low risk	Some concerns	Some concerns	Well-conducted RCT with adequate methods, limited by lack of protocol registration
Yoo et al. 2017 [35]	Crossover	Not applicable	Low risk	Low risk	Some concerns	Some concerns	Some concerns	Appropriate crossover methodology but limited reporting of methods
Acar et al. 2016 [51]	RCT	High risk	High risk	Low risk	High risk	Some concerns	High risk	Inadequate randomization and lack of blinding pose significant bias risks
Preston et al. 2016 [52]	RCT (pilot)	Low risk	Low risk	Some concerns	Low risk	Low risk	Some concerns	Well-designed pilot with good methodology, underpowered as acknowledged
Lazzari et al. 2015 [53]	RCT (double-blind)	Low risk	Low risk	Low risk	Low risk	Low risk	Low risk	High-quality double-blind RCT with appropriate methodology
Grecco et al. 2015 [54]	RCT (pilot)	Low risk	Low risk	Low risk	Low risk	Low risk	Low risk	High-quality pilot RCT with double-blind design and objective outcomes
Preston et al. 2014 [55]	Crossover (AB-BA)	Some concerns	Low risk	Low risk	Some concerns	Some concerns	Some concerns	Adequate crossover study but limited methodological reporting
Rostami et al. 2012 [56]	RCT	Some concerns	Some concerns	Low risk	Low risk	Some concerns	Some concerns	Adequate RCT methodology with some reporting limitations

Abbreviations: *CP:Cerebral Palsy;GMFCS:Gross Motor Function Classification System;ITT:Intention-to-Treat;LOCF:Last Observation Carried Forward;MACS:Manual Ability Classification System;NA:Not Applicable;RCT:Randomized Controlled Trial;TD:Typically Developing;tDCS:transcranial Direct Current Stimulation.*

Supplementary Table S6: Session-Response and Adherence.

Study	Planned Sessions	Actual Sessions (Mean)	Adherence (%)	Planned Duration (min)	Actual Duration (min)	Completion Rate (%)	Adherence Factors	Compliance Barriers	Session Intensity	Effect Size Category	Session-Effectiveness
Roberts et al. 2025 [43]	10	10	100	3600	3600	100	Structured camp setting, high motivation	Pre-intervention injury	High	Small	Moderate
Saussez et al. 2023 [44]	10	10	95	5400	~5130	95	Day-camp structure, family support	Epileptic seizure, behavioral issues	Very High	Moderate	High
Roostaei et al. 2023 [14]	12	12	100	720	720	100	Single-case design, individual attention	None	Moderate	Moderate	High
Fu et al. 2022 [45]	48	48	100	2400	2400	100	Hospital-based program, structured protocol	None	Very High	Large	Very High
Roberts et al. 2020 [46]	10	NR	97	300	~291	97	Camp setting, peer interaction	Transportation difficulties	Moderate	Large	Very High
Bortone et al. 2020 [47]	8	NR	87.5	480	~420	87.5	Individual sessions, novel technology	Study abandonment	Moderate	Small	Low
Decavele et al. 2020 [48]	24	18.8	78	480	375	84	Individualized games, therapist flexibility	Technical difficulties, motivational issues	Moderate	Large	High
Gagliardi et al. 2018 [49]	18	17.9	99.3	540	537	100	Clinical setting, structured protocol	None significant	Moderate	Small	Moderate
El-Shamy et al. 2018 [50]	36	36	100	1620	1620	100	Hospital-based, family commitment	None	Very High	Large	Very High
Yoo et al. 2017 [35]	2	2	100	60	60	100	Single-session crossover, research setting	None	Very Low	Small	Low
Acar et al. 2016 [51]	12	12	100	540	540	100	Clinical setting, engaging games	None	Moderate	Moderate	High
Preston et al. 2016 [52]	42	14	33	1260	420	75	Home-based flexibility	Equipment issues, busy schedules, motivation	Low	None	Very Low
Lazzari et al. 2015 [53]	1	1	100	20	20	100	Single session, research protocol	None	Very Low	Small	Moderate
Grecco et al. 2015 [54]	10	NR	95	200	~190	95	Short-term intensive protocol	Respiratory illness	Low	Large	Very High
Preston et al. 2014 [55]	56	NR	92	1680	~1546	92	School-based integration	Attendance issues	High	Small	Low
Rostami et al. 2012 [56]	12	12	100	1080	1080	100	Structured clinical protocol	None	High	Very Large	Very High
**SESSION CATEGORY:**											
**Very Low**	1-2 sessions	20-60 minutes total	100% (avg)	2 studies	22 participants	Varied	Limited data	Varied	Very Low	Small	Suitable for proof-of-concept or single-session interventions
**Low**	10-14 sessions	200-420 minutes total	64% (avg)	2 studies	36 participants	Varied	Variable	Varied	Low	None to Large	May be effective with intensive short-term protocols
**Moderate**	8-18 sessions	300-720 minutes total	91% (avg)	6 studies	136 participants	Varied	Generally positive	Varied	Moderate	Small to Large	Optimal balance of effectiveness and feasibility
**High**	36-56 sessions	1080-1680 minutes total	97% (avg)	3 studies	74 participants	Varied	Excellent	Varied	High	Small to Very Large	Most effective for sustained improvements
**Very High**	48+ sessions	2400+ minutes total	98% (avg)	3 studies	133 participants	Varied	Excellent	Varied	Very High	Small to Large	Maximum effectiveness for complex interventions
**ADHERENCE FACTOR:**											
**Setting: Clinic-based**	10 studies	Varied	96%	Varied	Varied	Varied	Structured environment, professional supervision, equipment reliability	Transportation, scheduling conflicts	Varied	Varied	Generally high effectiveness
**Setting: Home-based**	1 study	Varied	33%	Varied	Varied	Varied	Convenience, family involvement	Technical support, motivation, distractions	Varied	Varied	Lower effectiveness
**Setting: School-based**	1 study	Varied	92%	Varied	Varied	Varied	Integration with routine, peer interaction	Curriculum constraints, staff training	Varied	Varied	Moderate effectiveness

Abbreviations: *CP:Cerebral Palsy;GMFCS:Gross Motor Function Classification System;min:minutes;NR:Not Reported;VR:Virtual Reality.*

Supplementary Table S7: Grade Evidence Quality Assessment.

Outcome Domain	Evidence Base	Studies (n)	Participants (N)	Effect Sizes (n)	Study Designs	Risk of Bias	Inconsistency (I^2)	Indirectness	Imprecision	Publication Bias	Other Considerations	GRADE Quality	Effect Estimate [95% CI]	Interpretation
**PRIMARY META-ANALYSIS (RCT-Only):**														
Overall Motor Function	RCT primary analysis	5	190	40	All RCTs (5/5)	Serious (-1)*	Not serious (I^2=74% explained by moderators)	Not serious	Serious (-1)†	Not serious‡	Large moderation by age, technology, comparison	⊕⊕⊖⊖ **LOW**	SMD 0.41 [0.16, 0.66], p=0.001	Moderate effect; benefits exist but quality limited by RoB and sample size
Upper Limb Function	RCT subset analysis	4	130	20	All RCTs (4/5 included UL)	Serious (-1)	Not serious (I^2=77% expected)	Not serious	Serious (-1)	Not serious	Consistent positive direction	⊕⊕⊖⊖ **LOW**	SMD 0.59 [0.30, 0.88], p<0.001	Moderate-large effect; limited by RoB and N<400 OIS
Gross Motor Function	RCT subset analysis	1	60	6	Single RCT (Fu 2022)	Serious (-1)	Not assessable (single study)	Not serious	Very serious (-2)§	Not assessable	Single study limits generalizability; wide CI	⊕⊖⊖⊖ **VERY LOW**	SMD 1.52 [0.99, 2.05], p<0.001	Large effect but very low certainty; single-study evidence only
Balance Outcomes	RCT subset analysis	0	0	0	No RCTs with balance data	Not assessable	Not assessable	Not assessable	Not assessable	Not assessable	Insufficient data for RCT-only analysis	**Insufficient evidence**	Not estimable	Cannot rate; no RCT balance data extractable
Walking Capacity	RCT subset analysis	1	98	2	Single comparison (Roberts/Saussez camp studies)	Serious (-1)	Not assessable	Serious (-1)‖	Very serious (-2)	Not assessable	Camp-based setting may differ from clinic	⊕⊖⊖⊖ **VERY LOW**	SMD 0.14 [-0.25, 0.53], p=0.479	No significant effect; very low certainty
Functional Activities	RCT subset analysis	3	100	12	3 RCTs (Roberts, Saussez, Acar)	Serious (-1)	Not serious (I^2=55%)	Not serious	Serious (-1)	Not assessable	Heterogeneous outcome measures (GAS, COPM, etc.)	⊕⊕⊖⊖ **LOW**	SMD 0.13 [-0.18, 0.44], p=0.413	No significant effect; low certainty
**QUALITATIVE SYNTHESIS (All Included Studies):**														
Comprehensive Evidence Across All Domains	Mixed-design evidence base	16	397	Not pooled	12 RCTs, 2 crossover trials, 1 single-case design, 1 pre-post study	Very serious (-2)¶	Serious (-1)**	Not serious	Serious (-1)	Not serious	High heterogeneity; mixed designs preclude pooling; informative for context	⊕⊖⊖⊖ **VERY LOW**	Direction: positive in 14/16 studies (87.5%)	Qualitative synthesis shows consistent positive direction but very low certainty; not quantitatively pooled

Abbreviations: *CI, confidence interval; CP, cerebral palsy; GRADE, Grading of Recommendations Assessment, Development and Evaluation; I^2, I-squared heterogeneity statistic; n, number of studies; N, number of participants; OIS, optimal information size; p, probability value; Q, Cochran's Q test statistic; R^2, proportion of variance explained; RCT, randomized controlled trial; RoB, risk of bias; SMD, standardized mean difference; VR, virtual reality.*

Supplementary Table S8: Publication Bias Assessment.

**Assessment Method**	**Test Statistic**	**P-value**	**Interpretation**	**Conclusion**
**FUNNEL PLOT VISUAL INSPECTION:**				
Symmetry around pooled estimate	Visual assessment	N/A	Symmetric distribution; no obvious gaps	No visual evidence of bias
Precision-effect relationship	Appropriate funnel shape	N/A	Studies distributed appropriately by standard error	Consistent with absence of small-study effects
**STATISTICAL TESTS FOR SMALL-STUDY EFFECTS:**				
Egger's regression test	$t = 0.73$	0.470	Non-significant; no intercept different from zero	No bias detected
Begg's rank correlation	$\tau = 0.07$	0.623	Non-significant; no rank correlation with variance	No bias detected
Peters' regression test	$t = 0.58$	0.589	Non-significant; robust alternative to Egger	No bias detected
**TRIM-AND-FILL ANALYSIS:**				
Number of studies imputed	0 studies	N/A	No missing studies estimated	No adjustment required
Adjusted pooled effect	SMD = 0.41	N/A	Identical to observed (no imputation)	Effect estimate unchanged
Impact on conclusion	—	—	Primary finding maintained	Robust to potential bias
**ASSESSMENT SUMMARY:**				
Overall publication bias risk	Multiple concordant tests	All $p > 0.05$	No evidence from any test method	Low risk of publication bias
Methodological note	5 RCTs included (N=190)	—	Small N limits power but multiple tests agree	Conservative conclusion

Section and Topic	Item #	Checklist item	Location where item is reported
**TITLE**			
Title	1	Identify the report as a systematic review.	Title page - "Systematic Review and Meta-Analysis
**ABSTRACT**			
Abstract	2	See the PRISMA 2020 for Abstracts checklist.	Abstract section (structured)
**INTRODUCTION**			
Rationale	3	Describe the rationale for the review in the context of existing knowledge.	Introduction, paragraphs 1-3
Objectives	4	Provide an explicit statement of the objective(s) or question(s) the review addresses.	Introduction, final paragraph
**METHODS**			
Eligibility criteria	5	Specify the inclusion and exclusion criteria for the review and how studies were grouped for the syntheses.	Methods section 2.3 "Eligibility Criteria"
Information sources	6	Specify all databases, registers, websites, organisations, reference lists and other sources searched or consulted to identify studies. Specify the date when each source was last searched or consulted.	Methods section 2.2 "Search Strategy and Information Sources"
Search strategy	7	Present the full search strategies for all databases, registers and websites, including any filters and limits used.	Methods section 2.2 "Search Strategy and Information Sources"
Selection process	8	Specify the methods used to decide whether a study met the inclusion criteria of the review, including how many reviewers screened each record and each report retrieved, whether they worked independently, and if applicable, details of automation tools used in the process.	Methods section 2.4 "Study Selection and Data Collection"
Data collection process	9	Specify the methods used to collect data from reports, including how many reviewers collected data from each report, whether they worked independently, any processes for obtaining or confirming data from study investigators, and if applicable, details of automation tools used in the process.	Methods section 2.4 "Study Selection and Data Collection"
Data items	10a	List and define all outcomes for which data were sought. Specify whether all results that were compatible with each outcome domain in each study were sought (e.g. for all measures, time points, analyses), and if not, the methods used to decide which results to collect.	Methods section 2.4 "Study Selection and Data Collection"
	10b	List and define all other variables for which data were sought (e.g. participant and intervention characteristics, funding sources). Describe any assumptions made about any missing or unclear information.	Methods section 2.4 "Study Selection and Data Collection"
Study risk of bias assessment	11	Specify the methods used to assess risk of bias in the included studies, including details of the tool(s) used, how many reviewers assessed each study and whether they worked independently, and if applicable, details of automation tools used in the process.	Methods section 2.5 "Risk of Bias Assessment"
Effect measures	12	Specify for each outcome the effect measure(s) (e.g. risk ratio, mean difference) used in the synthesis or presentation of results.	Methods section 2.6 "Statistical Analysis and Evidence Synthesis"
Synthesis methods	13a	Describe the processes used to decide which studies were eligible for each synthesis (e.g. tabulating the study intervention characteristics and comparing against the planned groups for each synthesis (item #5)).	Methods section 2.6 "Statistical Analysis and Evidence Synthesis"
	13b	Describe any methods required to prepare the data for presentation or synthesis, such as handling of missing summary statistics, or data conversions.	Methods section 2.6 "Statistical Analysis and Evidence Synthesis"
	13c	Describe any methods used to tabulate or visually display results of individual studies and syntheses.	Methods section 2.6 "Statistical Analysis and Evidence Synthesis"
	13d	Describe any methods used to synthesize results and provide a rationale for the choice(s). If meta-analysis was performed, describe the model(s), method(s) to identify the presence and extent of statistical heterogeneity, and software package(s) used.	Methods section 2.6 "Statistical Analysis and Evidence Synthesis"
	13e	Describe any methods used to explore possible causes of heterogeneity among study results (e.g. subgroup analysis, meta-regression).	Methods section 2.6 "Statistical Analysis and Evidence Synthesis"
	13f	Describe any sensitivity analyses conducted to assess robustness of the synthesized results.	Methods section 2.6 "Statistical Analysis and Evidence Synthesis"
Reporting bias assessment	14	Describe any methods used to assess risk of bias due to missing results in a synthesis (arising from reporting biases).	Methods section 2.6 "Statistical Analysis and Evidence Synthesis"
Certainty assessment	15	Describe any methods used to assess certainty (or confidence) in the body of evidence for an outcome.	Methods section 2.6 "Statistical Analysis and Evidence Synthesis"
**RESULTS**			
Study selection	16a	Describe the results of the search and selection process, from the number of records identified in the search to the number of studies included in the review, ideally using a flow diagram.	Results section 3.1 "Study Selection and Characteristics" and Figure 1
	16b	Cite studies that might appear to meet the inclusion criteria, but which were excluded, and explain why they were excluded.	Results section 3.1 "Study Selection and

Section and Topic	Item #	Checklist item	Location where item is reported
			Characteristics" and Figure 1
Study characteristics	17	Cite each included study and present its characteristics.	Table 1 and Results section 3.1
Risk of bias in studies	18	Present assessments of risk of bias for each included study.	Results section 3.8 "Risk of Bias Assessment" and Supplementary Table 1
Results of individual studies	19	For all outcomes, present, for each study: (a) summary statistics for each group (where appropriate) and (b) an effect estimate and its precision (e.g. confidence/credible interval), ideally using structured tables or plots.	Table 2 and Results section 3.2 "Primary and Secondary Outcomes"
Results of syntheses	20a	For each synthesis, briefly summarise the characteristics and risk of bias among contributing studies.	Results section 3.3 "Meta-Analysis Results"
	20b	Present results of all statistical syntheses conducted. If meta-analysis was done, present for each the summary estimate and its precision (e.g. confidence/credible interval) and measures of statistical heterogeneity. If comparing groups, describe the direction of the effect.	Results section 3.3 "Meta-Analysis Results", Figure 2
	20c	Present results of all investigations of possible causes of heterogeneity among study results.	Results section 3.4 "Subgroup Analysis and Follow-up Effects" and Table 3
	20d	Present results of all sensitivity analyses conducted to assess the robustness of the synthesized results.	Results section 3.5 "Sensitivity Analysis" and Table 4
Reporting biases	21	Present assessments of risk of bias due to missing results (arising from reporting biases) for each synthesis assessed.	Results section 3.10 "Evidence Quality Assessment and Publication Bias", Figure 3, and Supplementary Table 4
Certainty of evidence	22	Present assessments of certainty (or confidence) in the body of evidence for each outcome assessed.	Results section 3.10 "Evidence Quality Assessment and Publication Bias" and Supplementary Table 2
**DISCUSSION**			
Discussion	23a	Provide a general interpretation of the results in the context of other evidence.	Discussion section, paragraphs 1-15
	23b	Discuss any limitations of the evidence included in the review.	Discussion section, paragraphs 16-19
	23c	Discuss any limitations of the review processes used.	Discussion section, paragraphs 16-19
	23d	Discuss implications of the results for practice, policy, and future research.	Discussion section, paragraphs 20-24
**OTHER INFORMATION**			
Registration and protocol	24a	Provide registration information for the review, including register name and registration number, or state that the review was not registered.	Methods section 2.1 "Study Design and Reporting Guidelines" - PROSPERO: CRD420251044140
	24b	Indicate where the review protocol can be accessed, or state that a protocol was not prepared.	Methods section 2.1 "Study Design and Reporting Guidelines"
	24c	Describe and explain any amendments to information provided at registration or in the protocol.	Not reported - no amendments made
Support	25	Describe sources of financial or non-financial support for the review, and the role of the funders or sponsors in the review.	End of manuscript - "Funding: Not applicable"
Competing interests	26	Declare any competing interests of review authors.	End of manuscript - "Competing interests: Not applicable"
Availability of data, code and other materials	27	Report which of the following are publicly available and where they can be found: template data collection forms; data extracted from included studies; data used for all analyses; analytic code; any other materials used in the review.	End of manuscript - "Acknowledgements: Not applicable"

From: Page MJ, McKenzie JE, Bossuyt PM, Boutron I, Hoffmann TC, Mulrow CD, et al. The PRISMA 2020 statement: an updated guideline for reporting systematic reviews. BMJ 2021;372:n71. doi: 10.1136/bmj.n71. This work is licensed under CC BY 4.0. To view a copy of this license, visit https://creativecommons.org/licenses/by/4.0/
